# Supplementary material for: Single-Cell Transcriptomic Profiling Unveils Dynamic Immune Cell Responses during Haemonchus contortus Infection
Source: Cells. 2024 May 15;13(10):842. doi: 10.3390/cells13100842 (PMC11120485; doi:10.3390/cells13100842)
Supplement: Supplementary file 1 [file cells-13-00842-s001.zip › cells-2983094-supplementary.pdf]

Table S1. Information of the cell suspension.

Sample Name	Concentration (number/uL)	Viability (%)
C-1	2,846	92%
C-2	3,158	90%
T-1	3,351	91%
T-2	3,021	94%

Table S2. Comparison before and after data quality control.

Sample name	Cells number before filtration	Median genes before filtration	Cells number after filtration	Median genes after filtration
C-1	7,334	1,382	7,107	1,394
C-2	8,345	1,346	8,041	1,357
T-1	7,692	1,356	7,418	1,366
T-2	8,385	1,358	7,865	1,371

Table S3. Proportion of different types of cells.

Cell type	Proportion of control group 1(%)	Proportion of control group 2(%)	Average proportion of control group (%)	Proportion of infection group 1(%)	Proportion of infection group 2(%)	Average proportion of infection group (%)
B	38.55%	30.60%	34.57%	33.73%	25.97%	29.85%
CD4 T	31.23%	36.39%	33.81%	31.46%	39.39%	35.43%
CD8 T	11.43%	11.89%	11.66%	16.97%	15.15%	16.06%
Mono	9.78%	10.79%	10.29%	5.56%	5.38%	5.47%
NK	3.25%	4.75%	4.00%	6.57%	8.66%	7.61%
DCs	1.23%	1.17%	1.20%	0.83%	0.76%	0.80%
Platelet	2.72%	2.27%	2.49%	2.88%	2.47%	2.67%
Plasmablast	1.81%	2.15%	1.98%	1.99%	2.23%	2.11%

Table S4. Sequences of oligonucleotide primers.

Primer name	Gene name	Sequence (5'-3')
β-actin-F	β-actin	CACCACACCTTCTACAAC
β-actin-R		TCTGGGTCATCTTCTCAC
IL-4-F	IL-4	GTACCAGCCACTTCGTCCAT
IL-4-R		GCTGCTGAGATTCCTGTCAA
IL-5-F	IL-5	CTGCCTATGTTTGTGCCAATGCT
IL-5-R		TTCCCATCACCTATCAGCAGAGTTTC
IL-6-F	IL-6	CTTCTGCTTTCCCTACCCCG
IL-6-R		AGTAGTCTGCTTGGGGTGGT
IL-10-F	IL-10	CCTTGTCGGAAATGATCCAG
IL-10-R		AGGGCAGAAAACGATGACAG
IL-13-F	IL-13	GTGTCATCCAAAGGACCAAGAGG
IL-13-R		TCGGATGTACTCACTGGAAACATG

Figure S1 Data filtering, dimensionality reduction and identification of highly variable genes

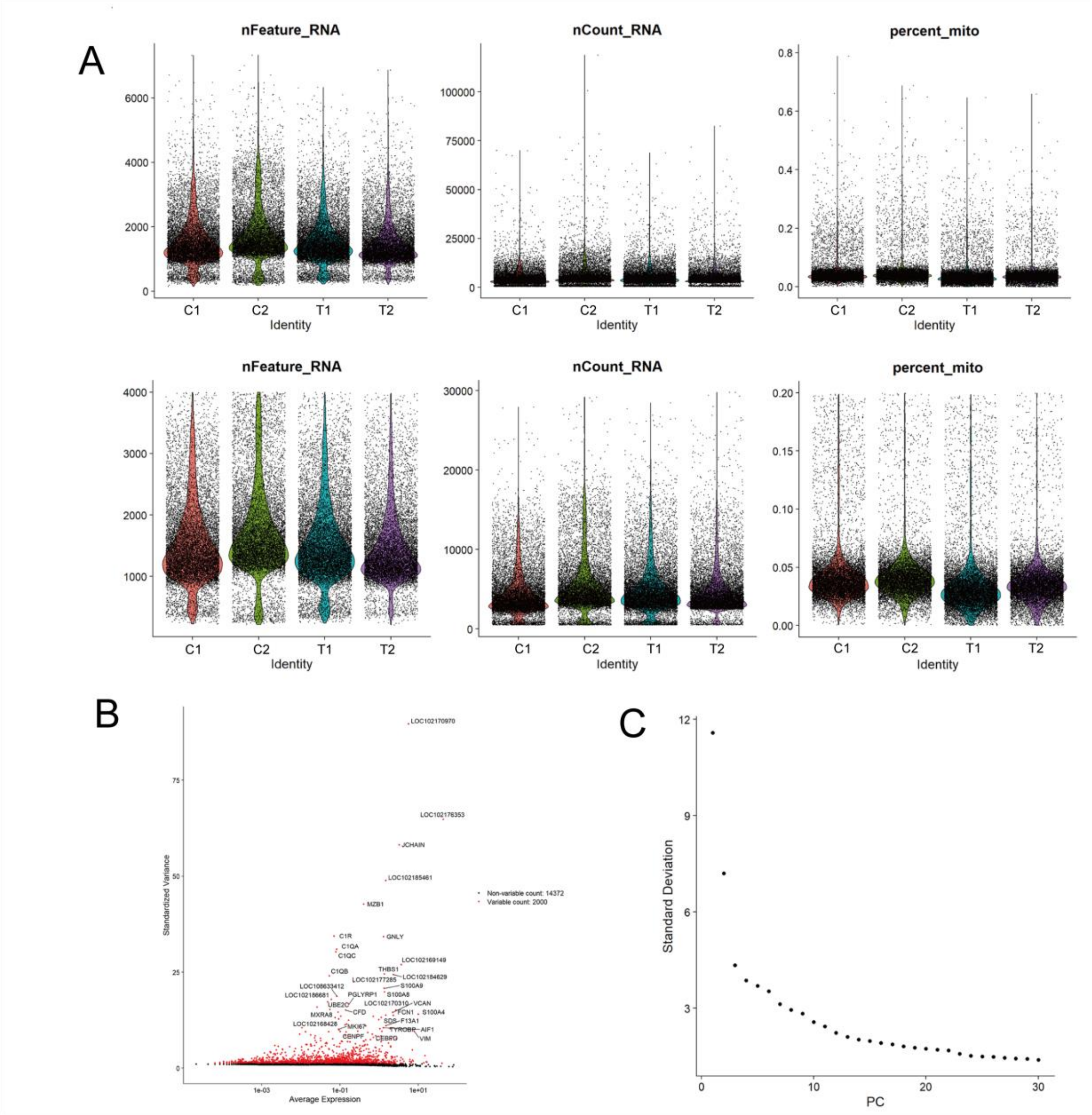

Figure S1. Data filtering, dimensionality reduction and identification of highly variable genes (A) Number of genes, counts, and mitochondrial gene ratio before and after data filtering; (B) The top 2000 highly variable genes; (C) The elbow plot.

Figure S2 The proportion of cell subpopulations and changes in cell cycle

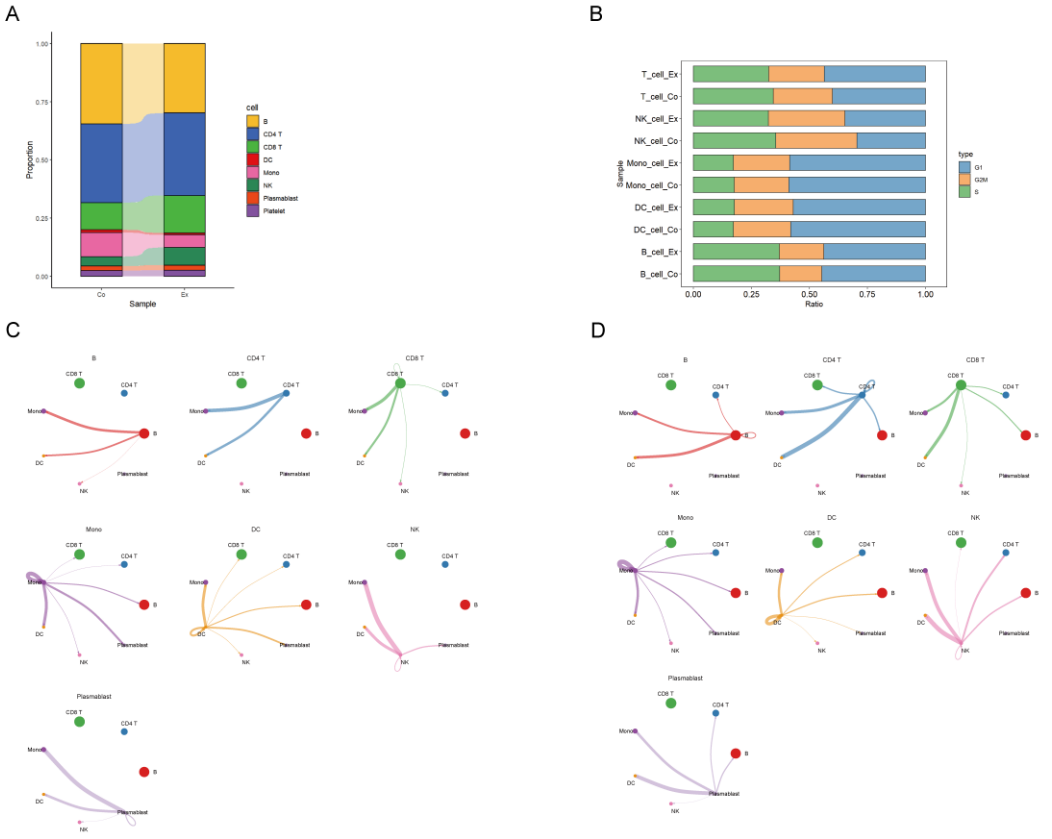

Figure S2. The proportion of cell subpopulations and changes in cell cycle. (A) Changes in the proportion of cell subpopulations; (B) The cell subpopulation cycle changes. (C) The number of cell communication in each subpopulation of the control group; (D) The number of cell communication in each subpopulation of the case group.
